# Dupilumab in Elderly Patients with Atopic Dermatitis—A Systematic Review and Meta-Analysis

**DOI:** 10.3390/biomedicines14010204

**Published:** 2026-01-17

**Authors:** Przemysław Hałubiec, Natalia Gołąbek, Anna Wojas-Pelc, Jacek Cezary Szepietowski, Andrzej Kazimierz Jaworek

**Affiliations:** 1 Department of Dermatology, Jagiellonian University Medical College, Botaniczna 3, 31-503 Cracow, Poland; anna.wojas-pelc@uj.edu.pl (A.W.-P.); andrzej.jaworek@uj.edu.pl (A.K.J.); 2 Department of Dermatology and Allergology, University Hospital, Botaniczna 3, 31-503 Cracow, Poland; 3 Doctoral School of Medical and Health Sciences, Jagiellonian University Medical College, Łazarza 16, 31-530 Cracow, Poland; 4 Student Scientific Group of Dermatology, Jagiellonian University Medical College, Botaniczna 3, 31-503 Cracow, Poland; natalia.golabek@student.uj.edu.pl; 5 Division of Dermatology, Venereology and Clinical Immunology, Faculty of Medicine, Wroclaw University of Science and Technology, Chałubińskiego 1, 50-368 Wroclaw, Poland; jacek.szepietowski@pwr.edu.pl; 6 Department of Dermato-Venereology, 4th Military Hospital, Weigla 5, 50-981 Wroclaw, Poland

**Keywords:** atopic dermatitis, dupilumab, elderly, quality of life, drug safety

## Abstract

**Background:** Atopic dermatitis (AD) is a chronic inflammatory skin disorder characterized by pruritic eczematous lesions that significantly alter quality of life of patients. Dupilumab, a new biologic agent, has demonstrated efficacy and safety in the general adult population with AD. However, evidence on its use in elderly patients is limited. **Objectives:** The objective of this work was to systematically assess the effectiveness and safety of dupilumab in patients aged ≥60 years with AD, based on published data. **Methods:** A systematic review and meta-analysis were conducted following the PICO(S) framework. Articles written in English and published before 31 December 2024 that investigated patients ≥ 60 years with AD treated with dupilumab were included. Meta-analysis of the observational studies was performed using a random-effects model with subgroup and meta-regression analyses. **Results:** Twenty-one articles met the inclusion criteria. After 16 weeks of treatment, dupilumab significantly reduced disease severity (EASI: 21.8; 95% CI: 18.3–25.2), intensity of pruritus (P-NRS: 5.8; 95% CI: 4.2–7.3), and quality of life impairment (DLQI: 11.3; 95% CI: 6.1–16.5); all *p* < 0.001. Meta-regression revealed previous treatment with cyclosporin A as a predictor of a poorer response to treatment. The generalized-prurigo phenotype was associated with worse control of pruritus. The most common adverse events were conjunctivitis, injection site reactions, and facial flushing. **Conclusions:** Dupilumab appears to be an effective and well-tolerated treatment for AD in elderly patients. More research is warranted to evaluate its long-term effectiveness and safety in this age group.

## 1. Introduction

Atopic dermatitis (AD) is a chronic, relapsing inflammatory skin disease characterized by intense pruritus and eczematous lesions, with age-dependent distribution patterns. Most individuals with AD report a personal or family history of atopy [1,2]. The global prevalence of AD is estimated to be 10–20% in children and 2–8% in adults. Traditionally, AD has been classified into infantile (<2 years), childhood (2–12 years), and adolescent–adult (>12 years), each with distinct lesion morphology and anatomical predilection [3,4].

Recently, AD in individuals aged ≥60 years has been recognized as a separate clinical variant [4]. In this population, comorbidities, polypharmacy, and an increased risk of infections require careful selection of therapy, with a focus on both efficacy and safety [5,6]. Older adults are underrepresented in clinical studies due to strict exclusion criteria, including comorbidities and laboratory abnormalities. There are also phenotype-related differences in AD that can affect eligibility and enrollment; for example, flexural dermatitis, included in the Hanifin–Rajka criteria, is less common in elderly patients. Therefore, data from the general adult population cannot be directly extrapolated to this group [7,8].

Standard management includes patient education, avoidance of triggers, regular emollient use, and topical anti-inflammatory therapies. In more severe cases, phototherapy or systemic medications may be required [4]. However, immunosuppressants such as cyclosporin A (CsA) are often contraindicated in older adults due to an unfavorable risk profile [5].

Dupilumab is a fully human monoclonal antibody targeting the interleukin (IL)-4 receptor α subunit, thereby inhibiting the IL-4 and IL-13 signaling pathways essential for the type 2 inflammatory response [5]. It was the first biologic approved for moderate-to-severe AD and has shown a favorable safety profile and rapid clinical improvement in adult patients [6]. Dupilumab has also shown efficacy in the treatment of moderate-to-severe asthma and uncontrolled chronic rhinosinusitis with nasal polyps, supporting its safety and effectiveness across different patient populations [9].

Despite its increasing clinical use, a comprehensive synthesis of its effectiveness and safety in patients aged ≥60 years remains lacking. This study aims to systematically review the evidence for dupilumab therapy in patients aged ≥60 years with AD and to meta-analyze the eligible study data.

## 2. Materials and Methods

### 2.1. Protocol and Registration

This systematic review and meta-analysis was conducted in accordance with the Preferred Reporting Items for Systematic Reviews and Meta-Analyses (PRISMA) guidelines [10] and was registered in the PROSPERO database (registration number CRD42023421711).

### 2.2. Search and Study Selection

Two independent reviewers systematically searched PubMed, Embase, and Scopus for relevant studies published before December 31, 2024, using the following search string: “dupilumab AND (‘atopic dermatitis’ OR eczema) AND (elderly OR age OR old OR geriatric)”. Manual screening of reference lists from eligible publications was also performed. Only articles published in English were included due to feasibility constraints, including a lack of capacity for reliable translation during full-text screening and data extraction [11].

An additional targeted gray literature search was carried out to minimize publication bias. The search included conference materials indexed in Embase as well as trials available at ClinicalTrials.gov and the World Health Organization International Clinical Trials Registry Platform. Google Scholar was searched to capture studies published in non-indexed journals.

Study eligibility was evaluated according to the PICO(S) framework [12]. We included studies that enrolled patients aged ≥60 years with a confirmed diagnosis of AD (P) who were treated with dupilumab (I). The age cutoff was selected based on the widely accepted definition of AD in older adults in the current literature [13,14,15]. Where applicable, comparators included placebo or guideline-based therapy (C). The primary outcomes were changes in quantitative measures of AD severity and the incidence of adverse events (O). Eligible study designs included randomized controlled trials (RCTs) and real-world prospective or retrospective studies (S). Case reports and case series were considered only for safety assessments, as they can provide valuable insights into rare, clinically relevant adverse reactions not typically captured in RCTs or observational studies. Study selection was performed independently by two reviewers and any disagreements were resolved by a third reviewer.

Data collection was carried out independently by two reviewers, and discrepancies were resolved by a third reviewer through assessment of the original source material. Extracted variables included study design, study quality, number of participants, demographic characteristics (age and sex), measures of AD severity and clinical response, as well as the type and frequency of adverse events associated with dupilumab treatment.

**Figure 1:**
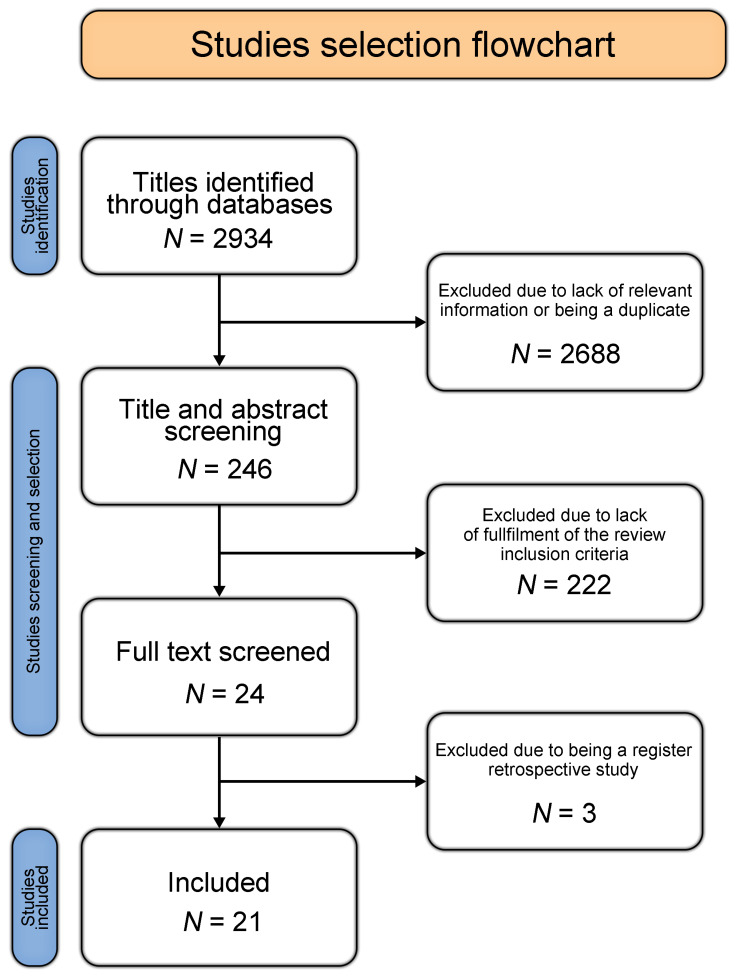
Studies selection flowchart.

Figure 1 presents a PRISMA flowchart summarizing the review process. The full protocol is available in the Supplementary Materials. The data extracted for this review are available from the corresponding author upon reasonable request.

### 2.3. Risk of Bias and Quality Assessment

The risk of bias in the included studies was evaluated using the RoB 2 tool for RCTs [16] and the ROBINS-I tool for nonrandomized intervention studies [17]. Study quality was further assessed using the NIH Study Quality Assessment Tool [18] (see Supplementary Figures S1 and S2). In addition, the GRADE approach was applied to the results from RCTs (Supplementary Table S1). All evaluations were conducted independently by two reviewers, with disagreements resolved by a third reviewer.

### 2.4. Meta-Analysis

No RCT exclusively investigating dupilumab in patients aged ≥60 years was identified. A pooled individual patient data analysis from Silverberg et al. [19], aggregating four RCTs, was included in the narrative synthesis only. Therefore, all meta-analytic estimates were derived from real-world observational studies.

Only studies reporting data at both baseline and week 16 were included in the quantitative synthesis. Outcomes included changes from baseline to week 16 in Eczema Area and Severity Index (EASI), Pruritus-Numerical Rating Scale (P-NRS), and Dermatology Life Quality Index (DLQI) scores. Mean changes from baseline and corresponding standard errors (SEs) were extracted. Where necessary, SEs for paired differences were calculated as follows:

$$SE=\sqrt{{SE}_{pre}^{2}+{SE}_{post}^{2}-2\times r\times {SE}_{pre}\times {SE}_{post}}$$

where *r* denotes the correlation coefficient between baseline and follow-up measures. As the actual values of *r* were unavailable, sensitivity analyzes were performed using *r* = 0.3, 0.5, and 0.7. Final estimates were based on *r* = 0.5 because it produced consistent results across all outcomes.

Meta-analyses were conducted using inverse-variance weighting under a random-effects model with τ^2^ estimated via restricted maximum likelihood. Confidence intervals for pooled effects were calculated using the Hartung–Knapp method to improve robustness in the presence of between-study heterogeneity. Forest plots were generated for each outcome.

Mean improvements were calculated as inverse-variance-weighted mean changes from individual studies.

Heterogeneity was assessed using τ^2^, I^2^ (considered substantial if >50%), and Cochran’s Q test (considered statistically significant at *p* < 0.10). Leave-one-out sensitivity analyses were performed by excluding each study in turn and recalculating the pooled estimate.

Subgroup analyses were conducted using the Q-test for subgroup differences (mixed-effects model), evaluating factors such as study location and study quality. Because preliminary analyses suggested two subgroups based on treatment response, we also performed an exploratory classification based on treatment response, defining “good response” as a mean change above the overall pooled estimate and “poor response” otherwise.

Univariate meta-regression analyses were performed using restricted maximum likelihood-based random-effects models with Hartung–Knapp adjustment, incorporating study-level clinical and methodological characteristics as potential effect modifiers.

Publication bias was visually assessed using funnel plots. Egger’s test was not performed due to the small number of included studies (*N* < 10) [20].

All analyses were conducted in R (version 4.4.1; R Core Team, 2024) using the *meta* package. Statistical significance was set at *p* < 0.05.

## 3. Results

### 3.1. Study Selection

The initial search yielded 2934 publications. After title screening, 246 records were selected for abstract review. Of these, 24 articles were identified for full-text review based on prespecified inclusion criteria. Three articles were subsequently excluded because they were retrospective registry analyses [21,22,23]. A search of the gray literature yielded no additional results.

Ultimately, 21 articles met the eligibility criteria (Table 1, Tables S2 and S3): a pooled analysis of RCTs reporting data for participants aged ≥60 years from the LIBERTY AD SOLO 1 and 2, LIBERTY AD CAFÉ, and LIBERTY AD CHRONOS studies [19]; a prospective study [24]; six retrospective observational studies [25,26,27,28,29,30], covering a total of 690 subjects; four case series and nine case reports [31,32,33,34,35,36,37,38,39,40,41,42,43], involving 26 subjects. Six case reports specifically described adverse drug reactions [31,34,35,36,37,39,40].

### 3.2. Summary of Included Studies

Among the eight clinical studies evaluated in this review, six met acceptable quality standards with no more than three negative ratings according to the NIH Study Quality Assessment Tool [19,24,25,27,29,30]. The GRADE evidence profile indicated a moderate certainty of evidence for the pooled RCTs analysis (Table S1).

Most of the participants were aged 65–80 years, and one study enrolled only individuals aged ≥80 years [24].

The follow-up time ranged from 16 to 104 weeks, with most quantitative data derived from observations after 16 weeks.

Dupilumab was administered every other week in 83.5% of patients. For 5.5% of the subjects, data on the dosage regimen were unavailable.

**Figure 2:**
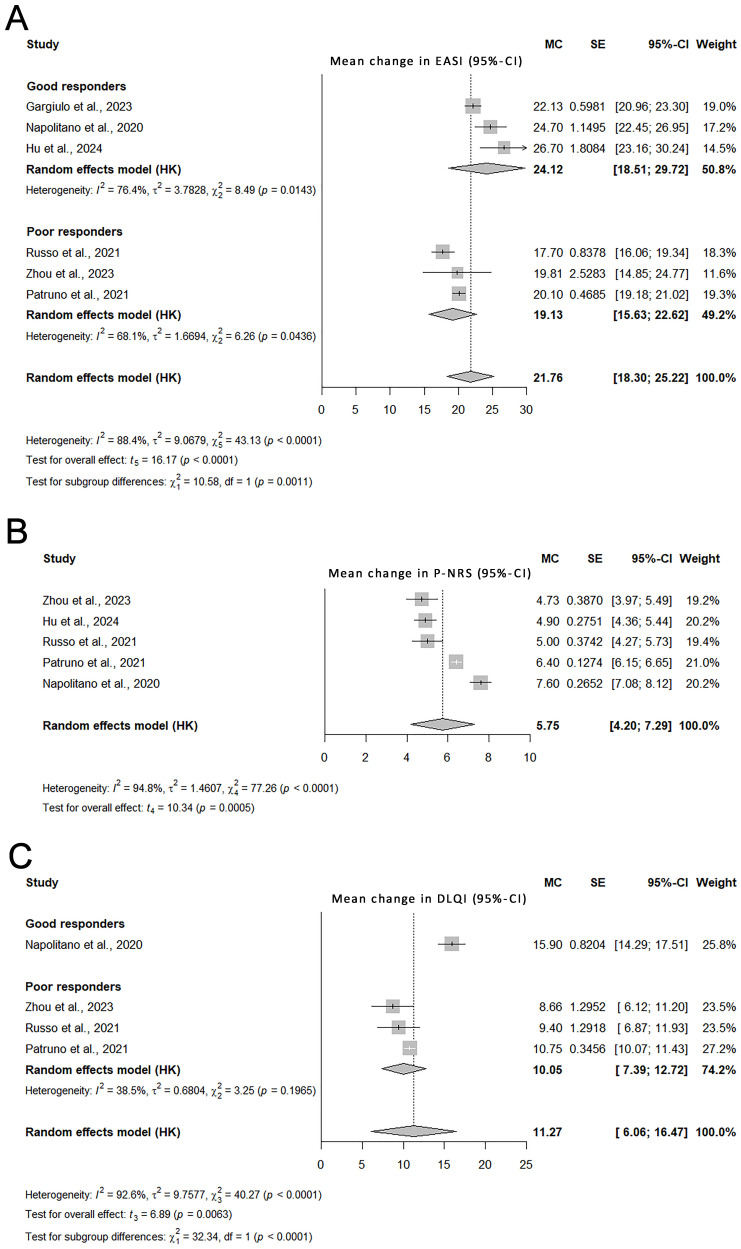
Forest plots from the meta-analysis of observational studies [24,25,26,27,29,30]. Data are presented as MC (SE) with 95% CI. The square size is proportional to the study weight. Subgroup analyses are presented when significant between-subgroup differences were detected. Pooled estimates are shown overall and by subgroup. Heterogeneity statistics are also displayed. (**A**) EASI, (**B**) P-NRS, (**C**) DLQI. Abbreviations: CI—confidence interval; DLQI—Dermatology Life Quality Index; EASI—Eczema Area and Severity Index; MC—mean change; P-NRS—Pruritus-Numerical Rating Scale; SE—standard error.

**Figure 3:**
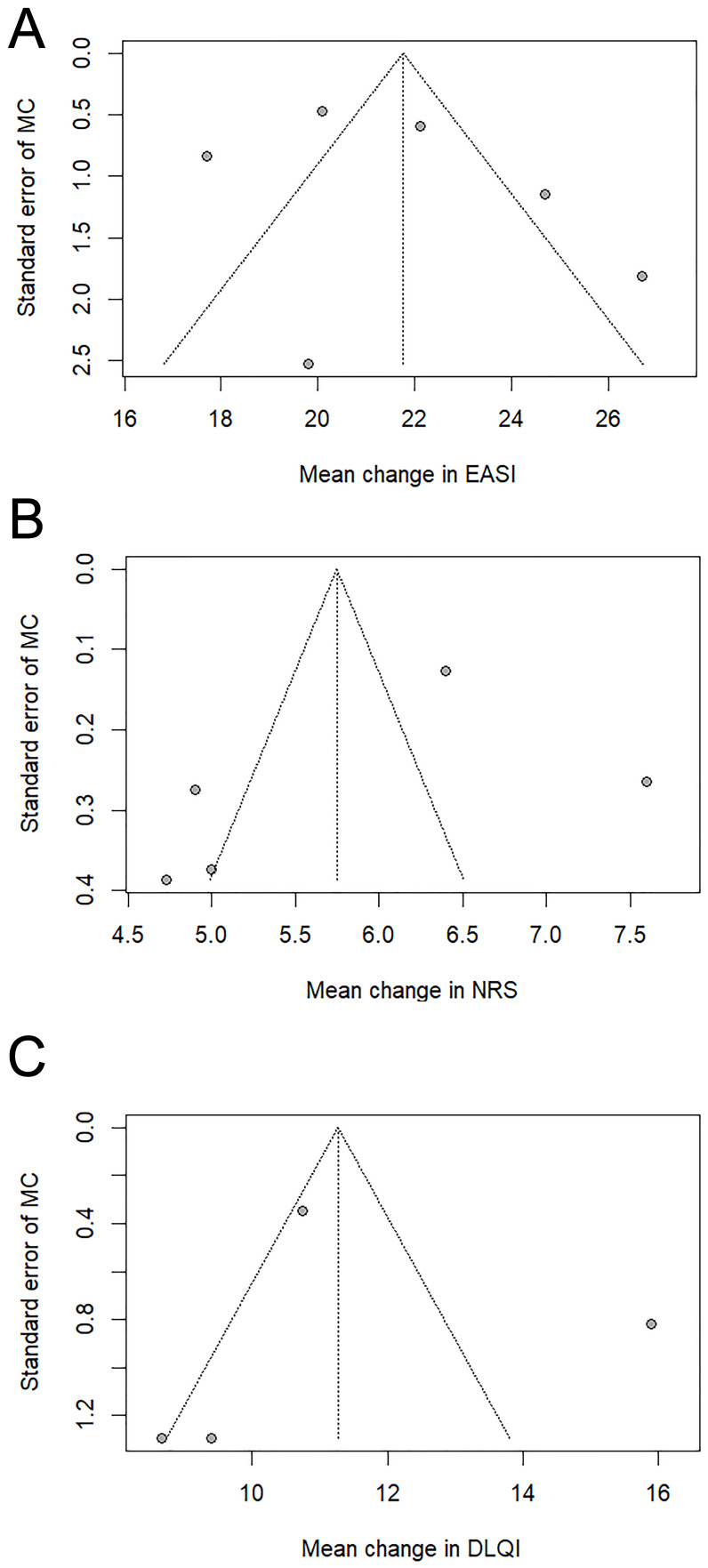
Funnel plots from the meta-analysis of observational studies. The 95% confidence intervals are shown for each plot. (**A**) EASI, (**B**) P-NRS, (**C**) DLQI. Abbreviations: CI—confidence interval; DLQI—Dermatology Life Quality Index; EASI—Eczema Area and Severity Index; MC—mean change; NRS—Numerical Rating Scale.

**Figure 4:**
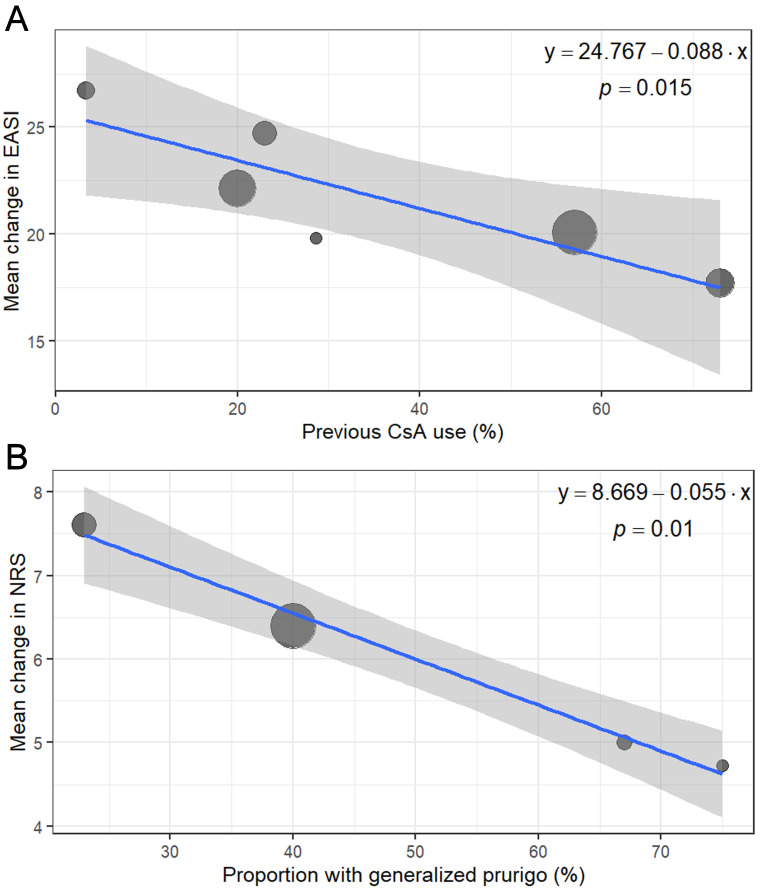
Bubble plots showing results of univariate meta-regression analyses from observational studies. (**A**) Proportion of subjects previously treated with CsA versus mean change in EASI. (**B**) Proportion of subjects with generalized-prurigo phenotype versus mean change in P-NRS. Associations are shown with regression lines and corresponding equations. The size of each bubble is proportional to the study weight. Abbreviations: CsA—cyclosporin A; EASI—Eczema Area and Severity Index; (P-)NRS—(Pruritus-)Numerical Rating Scale.

The results of the meta-analysis of real-world studies are presented in Figure 2. Funnel plots are presented in Figure 3. Statistically significant models from the univariate meta-regression are shown in Figure 4. Complete leave-one-out sensitivity analysis results are provided in Figure S3.

### 3.3. Severity of Skin Lesions

#### 3.3.1. Evidence from Randomized Controlled Trials

In the pooled RCTs analysis by Silverberg et al., the mean improvement in EASI at week 16 was 23.9 points (SE = 1.5) with weekly dupilumab dosing and 24.8 points (SE = 1.8) with every-other-week dosing, compared with 10.2 points (SE = 1.7) with placebo (*p* < 0.001 for both) [19].

#### 3.3.2. Evidence from Real-World Studies

The meta-analysis of real-world studies indicated a significant improvement in the EASI score by 21.8 points (95% CI: 18.3–25.2) after 16 weeks of treatment. The relative improvement in EASI was 79.7%. Substantial between-study heterogeneity was observed (I^2^ = 88.4%).

Sensitivity analyses confirmed that the pooled estimate remained stable after removal of any single study.

Univariate meta-regression demonstrated that a higher proportion of subjects previously treated with CsA was associated with a poorer response to dupilumab (*p* = 0.015, R^2^ = 0.88).

An exploratory analysis identified two response-based subgroups of studies (“good responders” and “poor responders”, as defined in the Section 2 Materials and Methods). The first subgroup included the studies by Hu et al., Gargiulo et al., and Napolitano et al. [25,26,30] and showed a pooled EASI improvement of 24.1 points (95% CI: 18.5–29.7). The second subgroup included the prospective study by Zhou et al. [24] and the studies by Patruno et al. and Russo et al. [27,29] and showed a pooled EASI improvement of 19.1 points (95% CI: 15.6–22.6) (*p* = 0.001 for between-subgroup differences). The subgroups contributed similar weights to the meta-analysis (50.8% and 49.2%).

These subgroups were consistent with the meta-regression findings, because the “poor responders” subgroup included studies with a higher proportion of patients previously treated with CsA.

Funnel plot assessment suggested potential publication bias.

### 3.4. Severity of Pruritus

#### 3.4.1. Evidence from Randomized Controlled Trials

The pooled RCTs analysis showed that at week 16, P-NRS improved by 3.8 points with every-other-week dosing and by 4.0 points with weekly dosing [19].

#### 3.4.2. Evidence from Real-World Studies

The overall improvement in P-NRS in real-world studies was 5.8 points (95% CI: 4.2–7.3), with a relative improvement of 71.6%.

Excluding any single study did not compromise the pooled estimate, supporting the robustness of the results.

In meta-regression, a higher proportion of the generalized-prurigo phenotype was associated with a smaller improvement in P-NRS at week 16 (*p* = 0.01, R^2^ = 0.99).

There was no statistically significant difference between the previously identified study subgroups (*p* = 0.57).

Funnel plot assessment suggested potential publication bias.

### 3.5. Quality of Life

#### 3.5.1. Evidence from Randomized Controlled Trials

Participants in the pooled RCTs analysis by Silverberg et al. had markedly lower baseline DLQI scores than participants in observational studies. In that analysis, DLQI improved by 9.4 points with every-other-week dosing and by 8.0 points with weekly dosing at week 16 [19].

#### 3.5.2. Evidence from Real-World Studies

The pooled DLQI improvement from real-world studies was 11.3 points (95% CI: 6.1–16.5). The relative improvement in DLQI in observational studies was 58.4%. The study by Napolitano et al. (the only study in the “good responders” subgroup with sufficient DLQI data) reported a significantly greater improvement than the “poor responders” subgroup (15.9 points [95% CI: 14.3–17.5] versus 10.1 points [95% CI: 7.4–12.7]; *p* < 0.001).

The pooled estimate remained stable in leave-one-out analyses.

Meta-regression did not identify any statistically significant predictors of DLQI improvement.

Funnel plot assessment suggested a low risk of publication bias.

### 3.6. Adverse Reactions

#### 3.6.1. Common Adverse Reactions in Included Studies

Among patients who received dupilumab every other week for 16 weeks (*N* = 617), the most common adverse reactions were conjunctivitis (6.2%), injection site reactions (2.6%), flushing (2.6%), fatigue (1.9%), and arthralgia (1.1%) (Table 2). Reaction severity was not reported.

In the pooled RCTs analysis, the adverse event rate per 100 patient-years (PY) was higher in the placebo group (40.9 events/100 PY) compared with dupilumab-treated patients (12.54 events/100 PY for weekly administration; 9.44 events/100 PY for every-other-week administration).

Weekly dosing of dupilumab was associated with a significantly higher incidence of injection-site reactions (*p* < 0.001) and conjunctivitis (*p* = 0.019) compared with every-other-week dosing [19].

#### 3.6.2. Rare Adverse Reactions Reported in Case Series and Case Reports

Among the case reports included in this review, five described rare adverse events potentially associated with dupilumab therapy. As these were isolated case reports, a causal relationship cannot be established.

These included a drug-induced sarcoid-like reaction in a 79-year-old patient [36] and the development of multiple psoriasiform plaques on various body sites in a 71-year-old man after two years of treatment [35]. In a separate case, a 66-year-old man developed insulin-requiring diabetes mellitus that resolved after discontinuation of dupilumab [31].

In another patient, the diagnosis of Sézary syndrome, presenting with erythroderma, was made two weeks after dupilumab initiation [39]. The second report described cutaneous T-cell lymphoma diagnosed nine weeks after dupilumab initiation [34]. In both lymphoma-related cases, pre-existing malignancy was not systematically ruled out by cutaneous biopsies with immunohistochemistry and T-cell receptor clonality assessment. Furthermore, the interval between treatment initiation and diagnosis of Sézary syndrome was short.

## 4. Discussion

### 4.1. General Considerations: Immunological and Clinical Context

AD in patients aged ≥60 years is increasingly recognized as a distinct clinical entity, driven by age-related changes in skin architecture and by immunosenescence [4]. Its management is influenced by factors such as comorbidities, polypharmacy, and patient dependence, which may limit therapeutic options [5].

Dupilumab selectively blocks the IL-4 receptor α subunit, reducing IL-4 and IL-13-mediated activation of the Janus kinase-signal transducer and activator of transcription signaling [44]. It is increasingly recognized as an effective treatment for other severe atopic diseases, such as type 2 asthma, with outcomes comparable to, or better than, those in the general population [45]. Similarly, it has been shown to be a safe and effective option for elderly patients with uncontrolled chronic rhinosinusitis with nasal polyps [46]. Other biologics are being investigated for use in older adults with AD. The most data are available for the anti-IL-13 antibodies tralokinumab [47] and lebrikizumab [48].

Structural and biochemical changes in aging skin include a decrease in epidermal thickness of 10–50%, reduced ceramide content, a decreased number of tight-junction proteins (occludin and claudins), and an increase in skin surface pH. Additionally, filaggrin synthesis decreases. Filaggrin is a cytoskeletal protein that binds and stabilizes keratin, and its proteolysis results in the release of highly hydrophilic amino acids, urocanic acid, and pyrrolidone carboxylic acid. Filaggrin is essential for both epidermal structure and hydration [13,49]. In physiologically aging skin, increased T_H_2 lymphocyte activity has also been observed [50].

In AD, these age-related skin changes are further amplified by excess IL-4 and IL-13, which promote T_H_2-driven inflammation and the recruitment of dendritic cells, eosinophils, and mast cells, collectively referred to as type 2 inflammation [51,52]. IL-4 also drives B-cell class switching toward IgE production, leading to further sensitization of mast cells and basophils. Key structural proteins, including filaggrin, loricrin, and involucrin, are downregulated, which, together with impaired ceramide synthesis, results in epidermal barrier dysfunction [53,54]. Antimicrobial peptides such as β-defensin 2, β-defensin 3, lipocalin 2, and cathelicidin LL-37 are likewise downregulated in response to IL-4 signaling, promoting dysbiosis characterized by *Staphylococcus aureus* predominance [55]. The itch–scratch cycle is exacerbated because IL-4 enhances C-C chemokine ligand (CCL) 17 and CCL22 production, facilitates IL-31 receptor signaling, increases peripheral sensory neuron sensitivity to pruritogens, and can directly activate sensory neurons [56,57]. Similar effects are attributed to IL-13, which downregulates essential epidermal structural proteins and lipids, promotes itch through transient receptor potential ankyrin 1-dependent pathways in sensory neurons, and enhances T_H_2 lymphocyte activity [52,58]. By blocking type 2 inflammation, dupilumab can reverse the IL-4- and IL-13-driven abnormalities described above. This reduces T_H_2-dependent inflammation and restores filaggrin and ceramide synthesis with improved skin barrier function. Furthermore, blockade of IL-4 signaling enhances synthesis of antimicrobial peptides, leading to normalization of the skin microbiota and reducing pruritus, thereby interrupting the itch–scratch cycle [52].

Older adults with AD are particularly burdened by comorbidities such as cardiometabolic conditions (including hypertension, heart failure, type 2 diabetes, obesity), dementia, chronic obstructive pulmonary disease, and malignancies [59,60,61]. These may increase patient functional dependence and interfere with dupilumab administration (for example, due to logistical barriers to attending clinic visits for medication dispensing or manual dexterity limitations with injections) [5,62]. However, such barriers could be appropriately addressed through health-system- and individual-level solutions.

Given its favorable safety profile, common comorbidities are generally not contraindications to dupilumab use in older adults. In patients at increased risk of nephrotoxicity or hepatotoxicity, dupilumab has been used with favorable outcomes [63,64].

Pleiotropic effects of dupilumab have been reported, including reduced expression of genes related to atherosclerosis and cardiovascular disease, further supporting its use in this population [65].

### 4.2. Dupilumab Effectiveness

The effectiveness of dupilumab in the general adult population is well established. A meta-analysis by Wu et al. showed that standard dupilumab therapy resulted in significantly greater improvements than placebo in EASI (by 10.9 points), P-NRS (by 9.3 points), and DLQI (by 9.7 points) (all *p* < 0.001) [66]. In a pooled analysis of four RCTs, Silverberg et al. reported even greater improvement in EASI (by 14.6 points) with comparable DLQI improvement (9.4 points) [19].

A meta-analysis of real-world studies in the general adult population reported relative improvements of 69.6% in EASI, 63.7% in P-NRS, and 67.7% in DLQI after 16 weeks of treatment [67]. In our meta-analysis of studies in older adults, similar outcomes were observed—EASI improved by 79.7%, P-NRS by 71.6%, and DLQI by 58.4%.

Most studies included in our analysis involved participants with similar age and disease severity profiles. An exception was the prospective observational study by Zhou et al. [24], which involved Chinese patients aged ≥80 years (predominantly male) with significantly higher baseline EASI scores than the other cohorts. Although these design differences might suggest that this study requires cautious interpretation, our analysis indicated that it corresponded with the studies by Patruno et al. and Russo et al. in terms of response to dupilumab [27,29].

These three studies comprised a subgroup characterized by a higher proportion of patients with prior CsA exposure and generalized-prurigo phenotype—both of which were associated with a reduced treatment response in meta-regression.

Previous treatment with immunosuppressive drugs (including CsA) was identified as the strongest baseline predictor of dupilumab nonresponse in a multivariable model (HR = 2.64; 95% CI: 1.10–6.37, *p* = 0.03), which also included sex, age at treatment initiation, body mass index, other allergic disorders, pruritus severity, and laboratory biomarkers in a long-term analysis of data from the Daily Practice BioDay Registry [68]. CsA exerts broad immunosuppressive effects by inhibiting calcineurin, thereby reducing IL-2 transcription and consequent T-cell activation. Treatment with CsA in patients with AD is associated with changes in T_H_2- and T_H_22-associated cytokines such as IL-13 and IL-22 [69]. There is also evidence that CsA might reduce IL-4- and IL-13-expressing CD4^+^ memory T cells [70]. This overlap in the mechanism of action may explain the association between prior CsA exposure and a poorer response to dupilumab.

The generalized-prurigo phenotype represents a chronic form of adult AD and might represent an overlap, coexistence, or a preceding phase between AD and prurigo nodularis, as these two disorders share many pathophysiological mechanisms, with the hallmark feature being chronic, severe, and debilitating pruritus of the skin [71]. The observed worse control of itch might be attributable to more pronounced skin diathesis in patients developing prurigo-like lesions [72]. Dupilumab may be less effective for itch driven by pruritogens other than IL-4 and IL-13, as suggested by studies comparing prurigo nodularis without AD features versus prurigo nodularis overlapping with AD [73,74].

Our findings do not allow firm conclusions about the optimal treatment duration of dupilumab therapy in elderly patients, since most included studies had follow-up limited to 16 weeks. Patruno et al. reported maximum clinical benefit at week 32, with preserved disease control through week 52 [28]. Similar observations were reported by Hu et al. [25]. Experimental data suggest that sustained depletion of sensitized T_H_2 lymphocytes may require at least 52 weeks of treatment [75].

Of note, the retrospective real-world studies in our analysis (with the exception of the study by Hu et al.) were conducted in Italy [26,27,29,30], which may limit the generalizability of the findings. However, RCTs in the general population have not demonstrated ethnicity-related differences in the clinical response to dupilumab [66], supporting the external validity of our meta-analysis.

### 4.3. Dupilumab Safety

In the general adult population, the largest increase in adverse event risk compared to placebo was observed for injection site reactions (RR = 2.24; 95% CI: 1.68–2.99), headache (RR = 1.47; 95% CI: 1.05–2.06), and conjunctivitis (RR = 2.64; 95% CI: 1.79–3.89) over follow-up periods ranging from 4 to 52 weeks [76].

The profile of adverse reactions was consistent across the studies included in this review. The administration of dupilumab every other week was as effective as weekly dosing, but with a significantly lower risk of adverse events [19]. The overall frequency of adverse reactions to dupilumab in the elderly population appears to be lower than in the general adult population.

Conjunctivitis is recognized as a characteristic adverse reaction to dupilumab in AD and is hypothesized to result from abnormalities in tear fluid production caused by blockade of IL-13 action with subsequent disruption of MUC5A synthesis [77] or increased *Demodex* activity [78].

Studies in the general adult population reported varying frequencies ranging from 10% to 50%, and it is consistently reported as the most common adverse reaction to dupilumab, with peak incidence after about 16 weeks of treatment [79,80,81]. According to VigiBase, in patients aged 0–44 years, the odds ratio of conjunctivitis was 28.1 (95% CI: 26.6–30.2), compared to 24.2 (95% CI: 22.6–25.9) in older patients [82], which is consistent with its relatively lower frequency observed in this study. Although this complication is usually mild to moderate, it remains the most common cause of dupilumab discontinuation.

It must be emphasized that currently available real-world data from studies in the general population highlight the presence of other ocular complications of dupilumab (such as pruritus, hyperemia, dryness, irritation, and increased lacrimation) that, together with conjunctivitis, are collectively described as dupilumab-associated ocular surface disease [83]. Therefore, its reported frequency may be influenced by factors such as prophylactic measures and follow-up duration. It cannot be excluded that some cases of dupilumab-associated ocular surface disease were reported as conjunctivitis in the included studies when other ocular symptoms were present.

Flushing has not been reported in RCTs. Its pathogenesis remains unclear and possible contributing factors include *Malassezia* and *Demodex* colonization, alcohol intake, or flares of rosacea, seborrheic dermatitis, and contact dermatitis. The etiology likely differs between patients [79]. The prevalence of facial erythema in real-world studies in the general population reaches 9%, with a discontinuation rate of 22% for dupilumab [84]. A systematic review by Jo et al. describes 101 cases in the literature with various clinical presentations of facial erythema and different treatment approaches. Approximately 11% of subjects discontinued treatment due to this complication [85]. Although this adverse reaction is considered one of the most characteristic of dupilumab treatment, proper reporting is difficult as it can be easily confused with flares of AD in the head and neck region.

Injection-site reactions include pain, swelling, and a burning sensation, and were reported in up to 30% of patients in a real-world study in the general adult population [86]. An analysis of data from the Food and Drug Administration Adverse Event Reporting System (FAERS) identified additional injection-site reactions such as injection-site dryness and eczema [87]. Most of these reactions are transient and self-limiting, requiring only simple supportive solutions.

One case of tuberculosis recurrence was reported after 4 months of treatment with dupilumab in a cohort of patients ≥ 80 years studied by Zhou et al.; however, no causal relationship can be established. Furthermore, age ≥ 75 years is itself a risk factor for tuberculosis recurrence [24,88].

Case series and case reports included in this review were evaluated primarily as sources of information on rare but potentially characteristic and clinically relevant adverse reactions to dupilumab. These events may not have been captured in clinical trials due to limited sample sizes and exclusion criteria.

Drug-induced sarcoidosis-like reactions are systemic granulomatous conditions that are clinically and histologically indistinguishable from sarcoidosis [89]. Tsitos et al. suggested that inhibition of IL-4 and IL-13 by dupilumab may disrupt the T_H_1/T_H_2 balance, skewing the immune response toward T_H_1 dominance and promoting granuloma formation, thus triggering a sarcoidosis-like reaction [36].

In another report, a patient developed psoriasiform eruptions on the scalp while experiencing complete remission of AD [40]. This “flip-flop” phenomenon has previously been described in the context of T_H_2-to-T_H_1 axis shift and emerging psoriasiform dermatitis in adults in the general population [90].

A case described by Blaylock et al. involved the onset of insulin-requiring diabetes mellitus shortly after dupilumab initiation [31]. The suspected mechanism involved the induction of anti-insulin autoantibodies. Notably, diabetes resolved after dupilumab discontinuation, supporting an association with that treatment.

Several case reports have raised concerns about a potential association between dupilumab and cutaneous T-cell lymphoma. However, the causal role remains doubtful, especially in view of the short latency periods observed between therapy initiation and lymphoma diagnosis. A pretreatment biopsy with immunophenotyping and T-cell receptor clonality assessment may be warranted in diagnostically uncertain cases [91].

Pharmacovigilance case-noncase disproportionality analyses in large databases (such as FAERS or VigiBase) could complement insights from case reports by assessing whether these incidentally reported reactions are disproportionately reported for dupilumab relative to background reporting rates.

### 4.4. Limitations

This work has several limitations. First, the search was restricted to articles published in English, which may introduce language bias. There was a considerable risk that publication bias influenced the results of our meta-analysis.

No RCT was specifically designed to assess the effectiveness and safety of dupilumab in patients aged ≥60 years.

Our quantitative synthesis was therefore based exclusively on observational studies, which were characterized by substantial heterogeneity in both design and patient characteristics. There were multiple potential sources of heterogeneity, including different inclusion criteria, baseline characteristics of the populations, and baseline disease severity. Interpretation of the meta-analysis requires acknowledging the relatively wide confidence intervals around the pooled estimate, rather than focusing only on the point estimate.

There is a risk of selection bias due to sampling participants who may not be representative of the target population. In particular, data on concomitant use of topical anti-inflammatory medications were not consistently reported or controlled across studies, and we could not include it as a confounder in sensitivity analyses. Channeling bias and indication bias may also have influenced these results, adversely affecting internal validity. External validity may be limited by the predominance of European (Italian) studies. Extrapolation to other populations may be constrained by differences in genetic background and healthcare systems. Analysis of drug survival and late-emergent adverse events was not possible due to limited data. Furthermore, characteristics typical of the elderly population (such as comorbidities, polypharmacy, dependence, and frailty) were underreported. Therefore, their potential confounding role could not be assessed.

We focused on the relatively short 16-week observation period because longer-term data were scarce. Therefore, it was not possible to assess long-term effectiveness, and there is a risk that some adverse reactions were not captured.

## 5. Conclusions

Findings from both RCTs and observational studies suggest that dupilumab is effective and safe for the treatment of AD in patients aged ≥60 years over a 16-week period. Although prior CsA exposure and a generalized-prurigo phenotype may be associated with an attenuated response, the overall clinical benefit of dupilumab remains substantial, supporting its use in older patients with AD.

For practicing physicians, the key take-home message is that age alone should not be considered a barrier to initiating treatment with dupilumab. In older adults, therapeutic decisions should be individualized, with particular attention to baseline disease phenotype, history of prior systemic therapy, comorbidities, functional status, and polypharmacy, and appropriate monitoring for adverse events and practical difficulties that may affect patients’ adherence.

Further research should assess long-term effectiveness and safety in this age group, ideally through randomized trials specifically designed for adults aged ≥60 years.

Future studies should also stratify outcomes by clinical phenotype and prior CsA exposure and systematically report key characteristics of the elderly population, including comorbidities, frailty, functional dependence, and polypharmacy, to provide a better foundation for patient-centered decision-making. Furthermore, greater attention is needed for standardized reporting of adverse events aligned with the Medical Dictionary for Regulatory Activities, severity grading metrics, and objective measures for some specific adverse reactions (such as tear film testing for dupilumab-associated ocular surface disease) or the potential immunogenicity of the dupilumab.

## Figures and Tables

**Table 1:** Summary of the main findings from studies assessing the effectiveness and safety of dupilumab for atopic dermatitis in patients aged ≥60 years. Additional data are provided in Table S3.

First Author	Study Type	Publication Year	Country	Participants (Male/Female)	Eligibility Criteria	Age (Years)	Dupilumab Regimen and Duration ^a^	Baseline EASI	EASI Change	Quality of Evidence and Risk of Bias	Reference
Silverberg	pooled analysis of four phase III, randomized, multicenter, double-blinded, placebo-controlled, parallel-group trials	2023	different countries in America, Asia and Europe	q2w: 54 (33/21) qw: 73 (45/28)	age > 60	q2w: 66 (63–72) qw: 64 (62–68) placebo: 65 (63–69)	total of 16w 300 mg s.c. every week (qw) or 300 mg s.c. every 2w (q2w) *versus* placebo ± TCS	qw: 29.3 (24.3–39.3) q2w: 29.9 (21.4–40.1) placebo: 27.8 (21.6–43.2)	LS mean change (SE) in EASI: qw: 23.9 (1.5) (*p* < 0.001) q2w: 24.8 (1.8) (*p* < 0.001) versus placebo: 10.2 (1.7)	fair quality, low risk of bias ^b^	[19]
Zhou	prospective observation	2023	China	28 (27/1)	age ≥ 80 and SCORAD ≥ 35	85.0 (3.6)	600 mg s.c. at first dose, then 300 mg s.c. every 2w total of 42w	40.5 (14.8)	16w: 20.7 (13.9), mean reduction in EASI 43.6%, EASI-50 was achieved in 53.9% (14/26)	fair quality	[24]
Hu	retrospective observation	2024	China	58 (28/30)	age ≥ 60	72.1 (9.5)	600 mg s.c. at first dose, then 300 mg s.c. every 2w for 52w ± TCS/TCI	33.9 (15.9)	16w: 7.2 (7.7) 28w: 6.8 (7.2) 52w: 4.8 (5.9)	fair quality	[25]
Gargiulo	retrospective observation	2023	Italy	40 (21/19)	age > 65	75.9 (6.5)	minimum of 16w; 30 participants for 32w; 23 participants for 52w; 8 participants > 104w; dosage not defined	25.3 (1.8)	16w: 3.2 (4.4) 32w: 1.8 (2.6) 52w: 1.6 (2.4) 104w: 0.9 (1.3)	low quality	[26]
Patruno	retrospective observation	2021	Italy	276 (159/117)	age > 65 and EASI ≥ 24	73.1 (6.8)	total of 16w; 600 mg s.c. at first dose then 300 mg s.c. every 2w ± TCS/TCI	29.2 (8.7)	16w: 9.1 (6.3)	fair quality	[27]
Patruno	retrospective observation	2021	Italy	105 (65/40)	age > 65 and EASI ≥ 24	70.8 (5.9)	total of 52w; 600 mg s.c. at first dose, then 300 mg s.c. every 2w ± TCS/TCI	not specified numerically	EASI reduction from baseline: 16w: 56% 32w: 84% 52w: 87%	low quality	[28] ^c^
Russo	retrospective observation	2021	Italy	26 (16/10)	age ≥ 65 EASI ≥ 24	68.4 (7.3)	total of 16w; 600 mg s.c. at first dose, then 300 mg s.c. every 2w	27.9 (4.5)	16w: 10.2 (4.0) (*p* < 0.001) reduction from baseline: 64.4%	fair quality	[29]
Napolitano	retrospective observation	2020	Italy	30 (17/13)	age ≥ 65 EASI ≥ 24	70.5 (4.5)	total of 16w; 600 mg s.c. at first dose, then 300 mg s.c. every 2w	27.2 (7.3)	16w: 2.5 (3.3)	fair quality	[30]

Continuous data are presented as mean (SD) or median (IQR [Q1–Q3]). ^a^ Concomitant use of TCS or TCI was mentioned in most studies, but without numerical data. Only Patruno et al. [27] reported exact baseline use of TCS and TCI (40.3% and 32.0%, respectively) and discontinuation rates at week 16 (58.8% and 13.6%, respectively). Russo et al. [29] reported that there was no topical anti-inflammatory therapy at baseline, whereas Zhou et al. [24], Gargiulo et al. [26], and Napolitano et al. [30] did not specify whether topical anti-inflammatory therapy was used. ^b^ As this was the only RCT (pooled analysis) and it raised no concerns according to the RoB 2 tool criteria, the risk-of-bias graph is not presented. No nonrandomized interventional comparative studies were available for assessment with the ROBINS-I tool. ^c^ Not included in the quantitative synthesis because exact EASI scores were unavailable. Abbreviations: EASI—Eczema Area and Severity Index, LS—least squares, RCT—randomized controlled trial, qw—dupilumab administered every week, q2w—dupilumab administered every two weeks, SE—standard error, SCORAD—Scoring Atopic Dermatitis, s.c.—subcutaneous, TCI—topical calcineurin inhibitors, TCS—topical corticosteroids, w—week(s).

**Table 2:** Adverse reactions to standard-dose dupilumab administered every other week for 16 weeks.

First Author	Number of Participants	Conjunctivitis	Injection Site Reaction	Flushing	Fatigue	Arthralgia	Nasopharyngitis	Headache	Generalized Lymphadenopathy	Alopecia	Herpes Infection	Other	Reference
Silverberg q2w ^a^	54	2 (3.7%)	-	-	1 (1.9%)	4 (7.4%)	3 (5.6%)	1 (1.9%)	-	-	2 (3.7%)	1 (1.9%) ^b^	[19]
Zhou	28	-	1 (3.6%)	-	-	-	-	-	-	-	-	3 (10.7%) ^c^	[24]
Hu	58	6 (10.3%)	1 (1.7%)	1 (1.7%)	-	-	-	-	-	-	-	6 (10.3%) ^d^	[25]
Gargiulo	40	-	-	-	-	-	-	-	-	-	-	-	[26]
Patruno	276	11 (4.0%)	8 (2.9%)	10 (3.6%)	8 (2.9%)	3 (1.1%)	-	2 (0.7%)	2 (0.7%)	-	-	-	[27]
Patruno	105	14 (13.3%)	6 (5.7%)	5 (4.8%)	3 (2.9%)	-	-	-	-	2 (1.9%)	-	-	[28]
Russo	26	3 (11.5%)	-	-	-	-	-	-	-	-	-	-	[29]
Napolitano	30	2 (6.7%)	-	-	-	-	-	-	-	-	-	-	[30]
Total	617	38 (6.2%)	16 (2.6%)	16 (2.6%)	12 (1.9%)	7 (1.1%)	3 (0.5%)	2 (0.3%)	2 (0.3%)	2 (0.3%)	2 (0.3%)	10 (1.6%)	

^a^ Data from the group treated with qw dupilumab (*N* = 73): injection site reaction: 19; nasopharyngitis: 11; conjunctivitis: 10; fatigue: 2; arthralgia: 1; headache: 4; upper respiratory tract infection: 4; herpes: 1; keratitis: 1. ^b^ 1 case of urinary tract infection. ^c^ 1 case of psoriasiform rash, 1 case of gastrointestinal discomfort, 1 case of recurrence of tuberculosis. ^d^ 6 cases of folliculitis.

## Data Availability

The raw data supporting the conclusions of this article will be made available by the authors on request.

## Supplementary Materials

The following supporting information can be downloaded at https://www.mdpi.com/article/10.3390/biomedicines14010204/s1, Figure S1: Studies quality assessment chart based on the NIH Study Quality Assessment Tool. Both randomized controlled trials (RCTs) and non-randomized studies are included. All assessment questions for both study types are listed. A green dot indicates fair quality, a red dot indicates poor quality, a yellow dot denotes that the information could not be determined, and a gray dot signifies that the question was not applicable. A study was considered of fair quality if no more than three items were rated as poor quality; Figure S2: Quality assessment chart for case series based on the NIH Study Quality Assessment Tool. A green dot indicates fair quality, a red dot indicates poor quality, a yellow dot denotes that the feature could not be determined, and a gray dot signifies that the item was not applicable. A case series was considered of fair quality if no more than one criterion was rated as poor quality; Figure S3: Graphical presentation of the leave-one-out sensitivity analysis results. The exclusion of any single study for any variable did not alter the overall interpretation of the meta-analysis outcomes; Table S1. GRADE evidence profile. The analysis of pooled data from RCTs by Silverberg et al. was assessed [19]; Table S2. Summary of case series and case reports evaluating dupilumab for AD in patients aged ≥60 years. If a report also included patients aged < 60 years, data for those patients are not presented; Table S3. Summary of findings from studies assessing the effectiveness and safety of dupilumab for atopic dermatitis in patients aged ≥60 years—complete data; Study Protocol.

## Abbreviations

The following abbreviations are used in this manuscript:

AD	Atopic dermatitis
CCL	C-C chemokine ligand
CsA	Cyclosporin A
DLQI	Dermatology Life Quality Index
EASI	Eczema Area and Severity Index
IL	Interleukin
PRISMA	Preferred Reporting Items for Systematic Reviews and Meta-Analyses
PY	patient-years
RCTs	randomized controlled trials
SE	standard error
(P-)NRS	(Pruritus-)Numerical Rating Scale
