# Supplementary material for: Dupilumab in Elderly Patients with Atopic Dermatitis—A Systematic Review and Meta-Analysis
Source: Biomedicines. 2026 Jan 17;14(1):204. doi: 10.3390/biomedicines14010204 (PMC12839263; doi:10.3390/biomedicines14010204)
Supplement: Supplementary file 1 [file biomedicines-14-00204-s001.zip › biomedicines-4074621-supplementary.pdf]

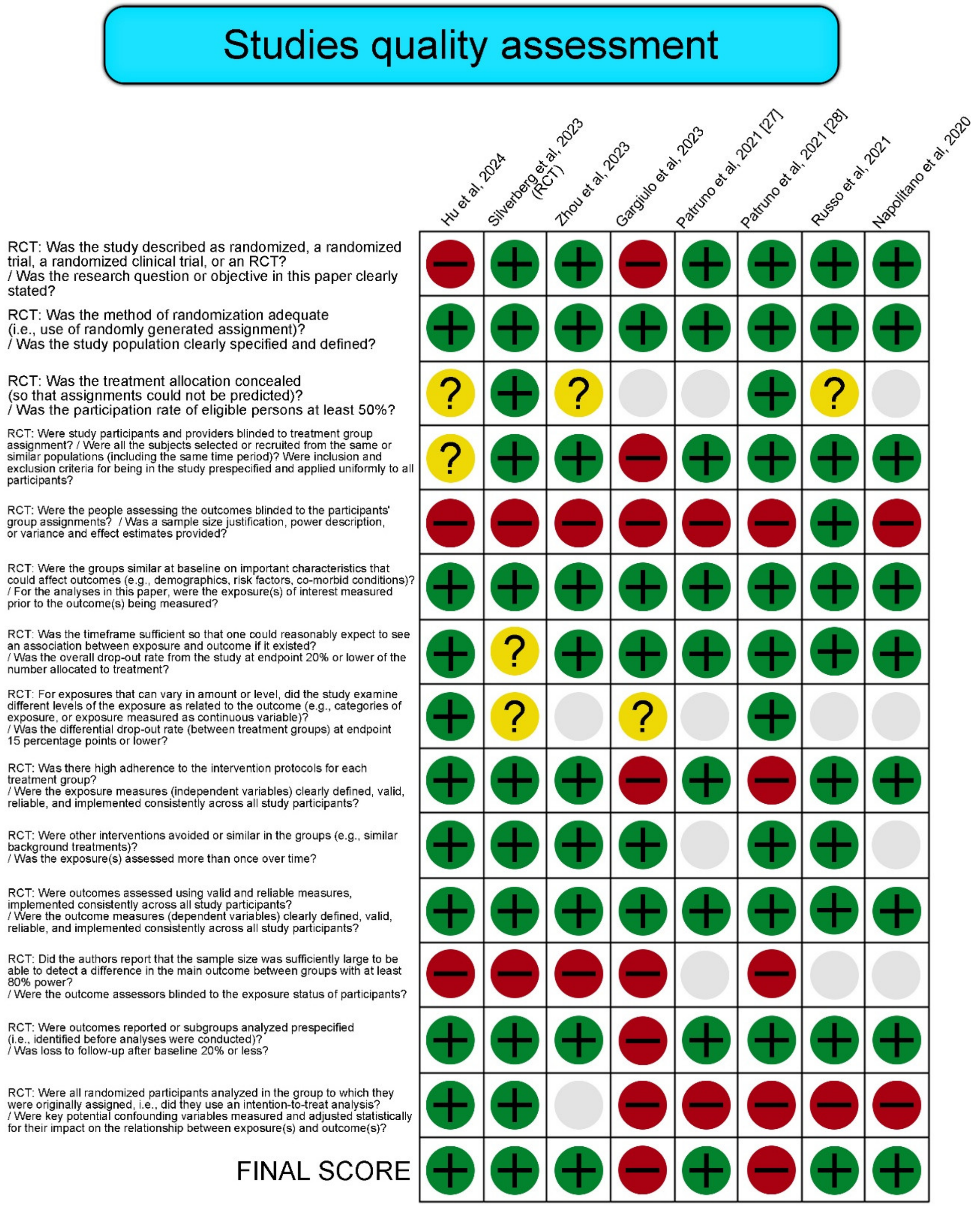

Supplementary Figure S1. Study quality assessment chart using the NIH Study Quality Assessment Tool. Both randomized controlled trials (RCTs) and non-randomized studies are included, and all assessment questions for both study types are shown. A green dot indicates fair quality, a red dot indicates poor quality, a yellow dot indicates that the information could not be determined, and a gray dot indicates that the question was not applicable. A study was considered fair quality if no more than three items were rated poor.

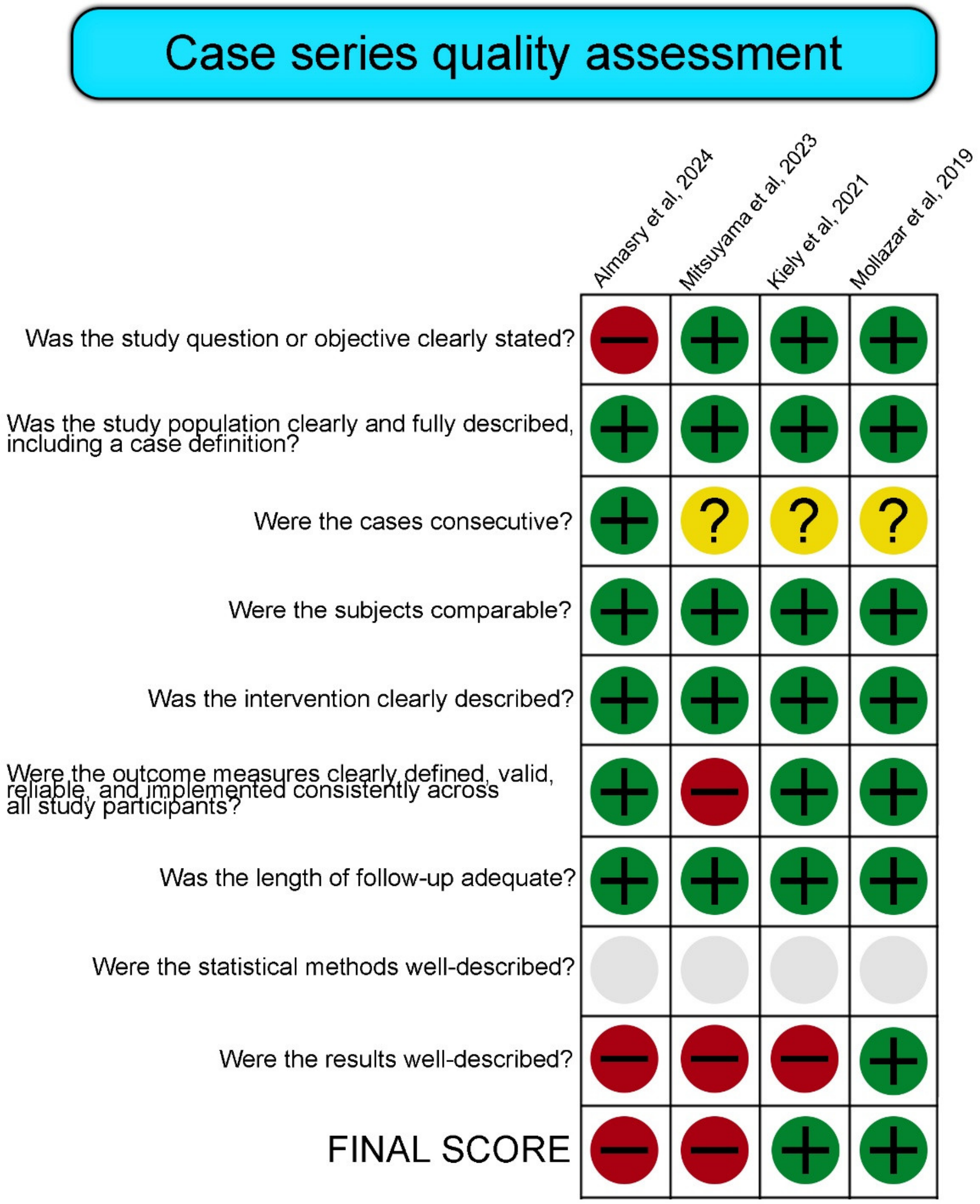

Supplementary Figure S2. Study quality assessment chart for case series using the NIH Study Quality Assessment Tool. A green dot indicates fair quality, a red dot indicates poor quality, a yellow dot indicates that the feature could not be determined, and a gray dot indicates that the item was not applicable. A case series was considered fair quality if no more than one criterion was rated poor.

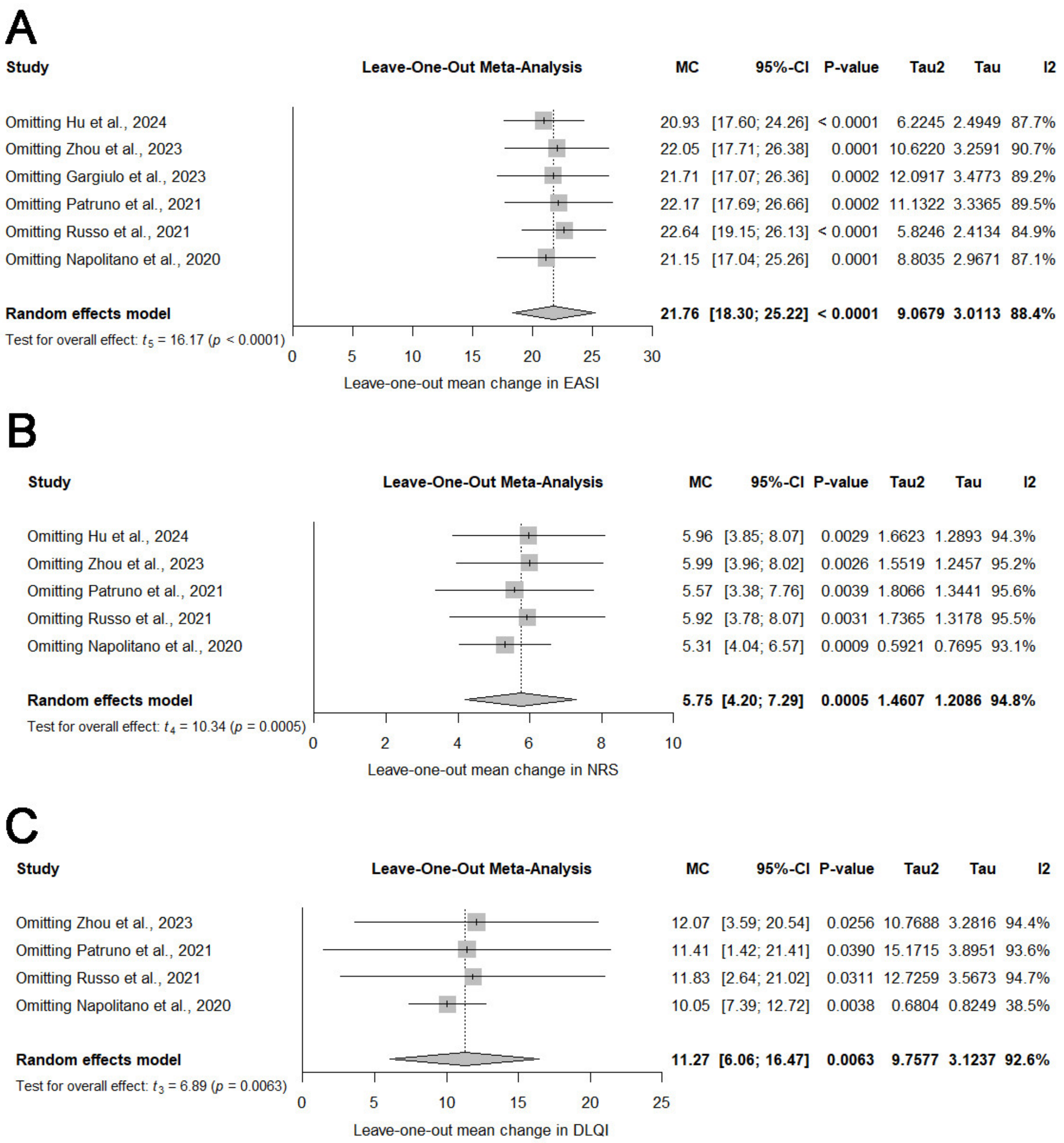

Supplementary Figure S3. Leave-one-out sensitivity analysis results. Excluding any single study, for any outcome, did not change the overall interpretation of the meta-analysis results.

Table S1. GRADE evidence profile. The analysis of pooled data from RCTs by Silverberg et al. was assessed [19].

Number of studies	Design	Risk of bias	Inconsistency	Indirectness	Imprecision	Other	Certainty (overall score)
Outcome: EASI score after 16 weeks of treatment with dupilumab in patients >60y with AD							
1	RCT	Low	n/a	Serious indirectness(↓)[a]	No serious imprecision	Publication bias(↓)[b] Large effect(↑)	⨁⨁⨁◯ **MODERATE**
Outcome: P-NRS score after 16 weeks of treatment with dupilumab in patients >60y with AD							
1	RCT	Low	n/a	Serious indirectness(↓)[a]	No serious imprecision	Publication bias(↓)[b] Large effect(↑)	⨁⨁⨁◯ **MODERATE**
Outcome: DLQI score after 16 weeks of treatment with dupilumab in patients >60y with AD							
1	RCT	Low	n/a	Serious indirectness(↓)[a]	No serious imprecision	Publication bias(↓)[b] Large effect(↑)	⨁⨁⨁◯ **MODERATE**
Outcome: Adverse reactions after 16 weeks of treatment with dupilumab in patients >60y with AD							
1	RCT	Low	n/a	No serious indirectness	No serious imprecision	Publication bias(↓)[b]	⨁⨁⨁◯ **MODERATE**

[a] This study is a retrospective analysis of data from four RCTs, which had different criteria for concomitant treatments administered alongside with dupilumab.

[b] No additional RCTs addressing the review question were identified. The arrows (↑, ↓) indicate whether given feature raised or lowered confidence in the evidence.

Abbreviations: AD–atopic dermatitis, GRADE–Grading of Recommendations, Assessment, Development, and Evaluations, n/a–not applicable, RCT–randomized controlled trial.

Table S2. Summary of case series and case reports evaluating dupilumab for AD in patients aged ≥ 60 years. If a report also included patients aged < 60 years, data for those patients are not presented.

First author	Publication year	Country	Number of participants (male/female)	Participants (male/female)	Dupilumab regimen and duration	Initial AD severity	Dupilumab's efficiency	Adverse reactions	Comments	Reference
Blaylock	2024	USA	1/0	66	600 mg s.c. at first dose, then 200 mg every two weeks for 7 months	palmar surfaces affected bilaterally	clinical remission	diabetes mellitus	suggested mechanism of diabetes mellitus includes formation of antibodies against endogenous insulin	[31]
Almasry	2024	Egypt	4/5	78<br>62<br>84<br>67<br>81<br>63<br>85<br>64<br>83	600 mg s.c. at first dose, then 300 mg every two weeks for 16 weeks	SCORAD/DLQI:<br>42.5/18<br>35.3/15<br>31.2/26<br>60.2/14<br>70.5/12<br>45.4/25<br>50.2/27<br>40.2/28<br>29.5/21	SCORAD/DLQI<br>19.7/9<br>16.3/7<br>15.1/10<br>32.9/5<br>35.2/5<br>25.0/10<br>29.6/12<br>20.5/14<br>15.2/12	one patient dropped the group due to the hypersensitivity drug reaction (anaphylaxis)	article considered as case series, not a study (as denoted by the journal)	[32]
Mistuyama	2023	Japan	3/1	65<br>69<br>71<br>84	600 mg s.c. at first dose, then 300 mg every week (qw) for 4-8 weeks	EASI:<br>16.3<br>29.0<br>20.7<br>17.3	2-4 weeks: significant improvement in pruritus<br>4-8 weeks: EASI-90	no serious adverse reactions	-	[33]
Park	2022	USA	1/0	72	9 weeks<br>dosage not defined	BSA: 12-15%	-	possible association with CTCL: mycosis fungoides stage IIA	a misdiagnosis of CTCL prior to dupilumab treatment cannot be ruled out	[34]
Phelps-Polirer	2022	USA	1/0	71	s.c. every 2 weeks for 2 years	-	10 weeks: significant improvement in AD symptoms;<br>3 months: clear skin,<br>IGA score: 1	2 years: multiple annular well-defined skin-colored plaques (forehead, back, bilateral upper, lower extremities)	-	[35]
Tsitos	2022	Germany	1/0	79	dupilumab s.c. every 2 weeks (+ 6 mg prednisolone p.o.) for 4 months	-	-	drug-induced neurosarcoid-like reaction	-	[36]
Kiely	2021	Ireland	2/0	65<br>69	300 mg every 2 weeks;<br>12 months, 15 months	IGA:<br>4<br>4	IGA after 1 month:<br>1<br>0	mild conjunctivitis (both cases)	-	[37]
Criado	2020	Brasil	1/0	87	600 mg s.c. at first dose, then 300 mg every 2 weeks (q2w);<br>16 weeks	VAS-P: 10	VAS-P: 2 after 1 month; "dramatic skin clearance after 4 months"	-	prurigo-nodularis phenotype	[38]

Tran	2020	USA	1/0	64	600 mg; 8 weeks	-	-	Sézary syndrome following the initiation of dupilumab;<br>2 weeks: erythrodermic rash (95% BSA)	-	[39]
Napolitano	2019	Italy	0/1	74	600 mg s.c. at first dose, then 300 mg every 2 weeks (q2w); 6 months	-	-	erythematous, hyperkeratotic plaques on the scalp	-	[40]
Yew	2019	Singapur (patient-China)	1/0	67	600 mg s.c. at first dose, then 300 mg every 2 weeks (q2w); 5 weeks	BSA: 15%, EASI: 12.9, DLQI: 21	week 4: BSA 10%, EASI 3.3, DLQI 13	-	-	[41]
Mollazar	2019	USA	2/0	60s (both)	600 mg s.c. at first dose, then 300 mg every 2 weeks (q2w); 9 months, 6 months	IGA:<br>4<br>4<br>NRSi:<br>7<br>10	IGA:<br>1<br>1<br>NRSi:<br>0<br>0	-	-	[36]
Treister	2018	USA	0/1	70s	600 mg s.c. at first dose, then 300 mg every 2 weeks (q2w); 8 weeks	IGA: 3<br>BSA: 50%	IGA treated (last office visit before discontinuation): 2, BSA treated: 15%; IGA after 8 weeks: 2, BSA after 8 weeks: 50%	-	-	[37]

In case series, the order of patients and their data was preserved as reported, where applicable.
Abbreviations**:** AD–Atopic Dermatitis, BSA–Body Surface Area, CTCL-cutaneous T-cell lymphoma, DLQI-Dermatology Life Quality Index, EASI–Eczema Area and Severity Index, IGA–Investigator's Global Assessment, n/d–no data, NRSi-Numerical Rating Scale Itch Intensity, qw–dupilumab administered every week, q2w–dupilumab administered every two weeks, s.c.–subcutaneous, SCORAD–Scoring Atopic Dermatitis, VAS–visual analogue scale.

Table S3. Summary of findings from studies assessing the effectiveness and safety of dupilumab for atopic dermatitis in patients aged ≥ 60 years – complete data.

First author	Year of publication	Number of participants	Disease onset	Clinical phenotype	Atopic comorbidities	Previous treatment	Initial severity of AD	16 week results	28-32 week results	52 week results	104 week results	Reference
Silverberg	2023	q2w: 54<br>qw: 73	duration of the disease:<br>q2w: 51 (20-62)<br>qw: 42 (14-61)	-	q2w:<br>allergic rhinitis – 24 (44%)<br>allergic asthma – 17 (31%)<br>conjunctivitis – 14 (26%)<br>food allergy – 10 (19%)<br><br>qw:<br>allergic rhinitis – 26 (36%)<br>allergic asthma – 24 (33%)<br>conjunctivitis – 15 (21%)<br>food allergy – 23 (32%)	-	q2w:<br>BSA: 56.3% (38.0-72.0)<br>EASI: 29.9 (21.4-40.1)<br>NRS: 8.0 (6.6-9.0)<br>SCORAD: 66.8 (60.0-78.3)<br>DLQI: 12.0 (8.0-19.0)<br><br>qw<br>BSA 52.0% (38.0-67.1)<br>EASI: 29.3 (24.3-39.3)<br>NRS: 7.9 (5.6-8.9)<br>SCORAD: 69.8 (56.5-75.8)<br>DLQI: 11.0 (7.0–17.0)	q2w:<br>EASI75: 63.0%<br>IGA 0-1: 44.4%<br>ΔNRS: -3.80<br>ΔDLQI: -9.36<br><br>qw:<br>EASI75: 61.6%<br>IGA 0-1: 39.7%<br>ΔNRS: -3.96<br>ΔDLQI: -7.99	-	-	-	[19]
Zhou	2023	28	onset:<br>18-59 y: 5 (17.7%)<br>>60 y: 23 (82.1)	flexural dermatitis – 15 (53.6%)<br>generalized eczema – 21 (75.0%)<br>head/neck – 14 (50.0%)<br>hand/foot – 14 (50.0%)	allergic rhinitis – 8 (28.6%)<br>allergic asthma – 2 (7.1%)<br>food allergy – 1 (3.6%)	traditional Chinese medicine – 16 (57.1%)<br>systemic CS – 13 (46.4%)<br>CsA – 8 (28.6%)<br>MTX – 1 (3.6%)<br>phototherapy – 3 (10.7%)<br>antihistamines – 15 (53.6%)<br>*Tripterygium wilfordii* – 6 (21.4%)	EASI: 40.52 (14.80)<br>NRS: 8.46 (1.05)<br>DLQI: 15.08 (7.09)<br>ADCT: 19.19 (2.95)	EASI: 20.71 (13.89)<br>NRS: 3.73 (2.36)<br>ΔNRS4: 26 (69.23%)<br>DLQI: 6.42 (6.59)<br>ADCT: 9.23 (6.05)<br>decrease in tIgE and eosinophil counts	-	-	-	[24]
Hu	2024	58	13.1 (17.3)	-	allergic rhinitis – 16 (27.6%)<br>allergic asthma – 13 (22.4%)	antihistamines – 82.8%<br>traditional Chinese medicine – 65.5%<br>systemic glucocorticoids – 3.4%	EASI: 33.9 (15.9)<br>PP-NRS: 8.2 (1.8)<br>SCORAD: 67.4 (19.7)<br>ADCT:	EASI: 7.2 (7.7)<br>PP-NRS: 3.3 (2.3)	EASI: 6.8 (7.2)<br>PP-NRS: 2.5 (2.1)	EASI: 4.8 (5.9)<br>PP-NRS: 2.1 (2.2)	-	[25]

						systemic immunosupressants – 3.4%	18.4 (4.2)<br>POEM:<br>18.6 (6.0)<br>DLQI:<br>10.5 (4.9)					
Gargiulo	2023	40	above 65 y in 75% of cases	flexural dermatitis – 19 (48%)<br>generalized eczema – 4 (10%)<br>prurigo nodularis – 6 (15%)<br>nummular eczema – 7 (18%)	allergic rhinitis – 8 (20%)<br>allergic asthma – 4 (10%)<br>conjunctivitis – 5 (13%)<br>food allergy – 5 (13%)	topical and systemic CS – 38 (95%)<br>cyclosporin A – 8 (20%)	EASI:<br>25.33 (1.83)	*N* = 40<br>EASI:<br>3.20 (4.35)<br>EASI75:<br>85.0%<br>EASI90:<br>57.5%<br>EASI100:<br>27.5%<br>ΔEASI:<br>-86.8%<br>vIGA0/1:<br>80.0%<br>ΔNRS4:<br>92.5%	*N* = 30<br>EASI:<br>1.56 (2.38)<br>EASI75:<br>96.7%<br>EASI90:<br>73.3%<br>EASI100:<br>53.3%<br>ΔEASI:<br>-92.2%<br>vIGA0/1:<br>80.0%<br>ΔNRS4:<br>93.3%	*N* = 23<br>EASI:<br>1.56 (2.38)<br>EASI75:<br>95.7%<br>EASI90:<br>73.9%<br>EASI100:<br>56.5%<br>ΔEASI:<br>-93.7%<br>vIGA0/1:<br>78.3%<br>ΔNRS4:<br>82.6%	*N* = 8<br>EASI:<br>0.87 (1.25)<br>EASI75:<br>100.0%<br>EASI90:<br>97.5%<br>EASI100:<br>62.5%<br>ΔEASI:<br>-96.5%<br>vIGA0/1:<br>100.0%<br>ΔNRS4:<br>100%	[26]
Patruno	2021	276	persistent: 71<br>late-onset: 205	flexural dermatitis – 125 (45%)<br>prurigo nodularis – 67 (24%)<br>head/neck eczema – 58 (21%)<br>generalized eczema – 43 (16%)<br>hand eczema – 43 (16%)<br>nummular eczema – 20 (7%)<br>erythroderma – 11 (4%)<br>>1 phenotype in 63 (23%) cases	allergic rhinitis – 47 (17%)<br>allergic asthma – 35 (13%)<br>conjunctivitis – 35 (13%)<br>food allergy – 10 (4%)	cyclosporin A – 158 (57%)<br>systemic CS – 115 (42%)<br>NB-UVB – 108 (39%)<br>MTX – 18 (7%)<br>omalizumab – 10 (4%)<br>apremilast – 9 (3%)<br>ustekinumab – 7 (3%)<br>other – 8 (3%)	EASI:<br>29.2 (8.7)<br>P-NRS:<br>8.9 (1.6)<br>S-NRS:<br>7.8 (1.8)<br>DLQI:<br>18.4 (4.7)	EASI:<br>9.1 (6.3)<br>P-NRS:<br>2.5 (2.4)<br>DLQI:<br>7.65 (6.4)	-	-	-	[27]
				flexural dermatitis with								

Patruno	2021	105	persistent: 29<br>late onset: 76<br><br>average disease duration:<br>19.97 (18.44) years<br>(1–70 years)	head/neck eczema –<br>21 (8%)<br>flexural dermatitis with hand eczema –<br>18 (7%)<br>flexural dermatitis with head/neck and hand eczema –<br>12 (4%)<br>flexural dermatitis –<br>11 (10%)<br>prurigo nodularis –<br>8 (8%)<br>hand eczema –<br>7 (7%)<br>generalized eczema –<br>6 (6%)<br>nummular eczema –<br>3 (3%)<br>flexural dermatitis + head/neck + hand eczema –<br>13 (12%)<br>flexural dermatitis + hand eczema –<br>12 (11%)<br>flexural dermatitis + head/neck eczema –<br>11 (10%)	allergic rhinitis –<br>21 (20%)<br>allergic asthma –<br>19 (18%)<br>conjunctivitis –<br>12 (11%)<br>food allergy –<br>5 (5%)	systemic CS –<br>96 (91%)<br>cyclosporin A –<br>83 (79%)<br>phototherapy –<br>72 (69%)<br>ustekinumab –<br>11 (10%)<br>omalizumab –<br>2 (2%)<br>MTX – 4 (4%)<br>azathioprine –<br>3 (3%)<br>MMF – 2 (2%)<br>apremilast –<br>1 (1%)<br>etanercept –<br>1 (1%)	-	ΔEASI:<br>-56.0%<br>ΔP-NRS:<br>-57.8%<br>ΔS-NRS:<br>-53.8%<br>ΔDLQI:<br>54.8%	ΔEASI:<br>-83.9%<br>ΔP-NRS:<br>-75.6%<br>ΔS-NRS:<br>-80.3%<br>ΔDLQI:<br>72.0%	ΔEASI:<br>-87.2%<br>ΔP-NRS:<br>-80.3%<br>ΔS-NRS:<br>-82.2%<br>ΔDLQI:<br>87.0%	-	[28]

				flexural + nummular dermatitis – 7 (7%)<br>flexural + nummular dermatitis + hand eczema – 7 (7%)<br>flexural dermatitis + prurigo nodularis – 6 (6%)<br>head/neck + hand eczema – 8 (8%)<br>prurigo nodularis + hand eczema – 4 (4%)<br>nummular dermatitis + hand eczema – 2 (2%)								
Russo	2021	26	average age of onset 47.1 (24.0)	prurigo nodularis – 6 (23%)<br>diffuse eczema – 14 (54%) with hand eczema in 7 cases (27%)<br>nummular eczema – 5 (19%),<br>psoriasiform pattern – 1 (4)	allergic rhinitis – 10 (39%)<br>allergic asthma – 4 (15%)<br>conjunctivitis – 3 (12%)	cyclosporin A – 19 (73%)<br>systemic CS – 24 (92%)<br>MTX – 2 (8%)<br>AZA – 1 (4%)<br>phototherapy – 13 (50%)	EASI: 27.9 (4.5)<br>P-NRS: 8.8 (1.0)<br>DLQI: 21.2 (5.5)	EASI: 10.2 (4.0)<br>P-NRS: 3.8 (2.2)<br>DLQI: 11.8 (7.3)<br>*P* <0.001 for each comparison<br><br>ΔEASI: -64.6%<br>ΔP-NRS: -56.8%<br>ΔDLQI: -41.4%	-	-	-	[29]

Napolitano	2020	30	persistent 8<br>late-onset 22<br>duration of the disease:<br>45.6 (11.6)	flexular dermatitis – 23 (77%)<br>prurigo nodularis – 5 (17%)<br>nummular eczema – 2 (7%)	allergic rhinitis – 4 (13%)<br>allergic asthma – 2 (7%)<br>conjunctivitis – 2 (7%)	systemic CS – 11 (37%)<br>cyclosporin A – 7 (23%)<br>UVB – 7 (23%)	EASI:<br>27.2 (7.3)<br>VAS-P:<br>8.5 (1.5)<br>DLQI:<br>20.9 (5.0)	EASI:<br>2.5 (3.3)<br>VAS-P:<br>0.9 (1.4)<br>DLQI:<br>5.0 (3.7)	-	-	-	[30]

The continuous data are shown as mean (SD) or as median (interquartile range Q1-Q3).

Abbreviations**:** AD–atopic dermatitis, ADCT–Atopic Dermatitis Control Tool, AZA–azathioprine, BSA–body surface area, CS–corticosteroids, DLQI–dermatology life quality index, EASI–Eczema Area and Severity Index, IGA–investigator's global assessment, MMF–mofetil mycophenolate, MTX–methotrexate, (NB-)UVB–(narrow-band) ultraviolet B, NRS–numerical rating score, qw–dupilumab administered every week, q2w–dupilumab administered every two weeks, SCORAD–scoring atopic dermatitis, tIgE–total immunoglobulin E, w–weeks, VAS–visual analogue scale, y–years.

STUDY PROTOCOL

ADMINISTRATIVE INFORMATION

TITLE

Dupilumab Effectiveness And Safety In Elderly Patients With Atopic Dermatitis - a protocol of a systematic review with meta-analysis – version 3.0

REGISTRATION

This systematic review was registered in PROSPERO under a number CRD42023421711.

AUTHOR RESPONSIBLE FOR THE PROTOCOL

Przemysław Hałubiec, MD
Chair of Dermatology, Jagiellonian University, Medical College
Doctoral School of Medical and Health Sciences, Jagiellonian University, Medical College
przemyslaw.halubiec@doctoral.uj.edu.pl
Botaniczna 3, 31-503 Cracow, Poland

AMENDMENTS

Amendment 1 (version 1.1)
Added April 23 2024
GRADE evidence profile was used to determine the quality of evidence from RCTs for particular outcomes (disease severity measured with EASI, itch severity measured with P-NRS, quality of life measured with DLQI, the adverse reactions to the treatment with dupilumab).

Amendment 2 (version 2.0)
Added June 15 2024
We re-evaluated possibility of conducting a meta-analysis based on the collected studies. We concluded that statistical synthesis of data from observational studies (referred also as "real-world studies") might be considered as feasible. All amendments to the protocol resulting from this decision are disclosed below and marked with "v2" sign. All articles published before 15th May 2024 were searched.

Amendment 3 (version 3.0)
Added February 1 2025
All amendments to the protocol resulting from this version are disclosed below and marked with "v3" sign. All articles published before 31th December 2024 were searched.

Amendment 4 (version 4.0)

Added January 6 2025

An additional targeted gray literature search was carried out to minimize publication bias. The search included conference materials indexed in Embase as well as trials available at ClinicalTrials.gov and the World Health Organization International Clinical Trials Registry Platform. Google Scholar was searched to capture studies published in non-indexed journals.

Amendments to the registry in the PROSPERO system:

they are reported here as these entries were impossible to update or correct in the PROSPERO system after the initial submission:

1) (v2) in entry 15. "Review question" the line "Comparator" is defined as "not applicable", which was intended to mean that the review did not intend to compare dupilumab to any other treatment. However, as we define in the subsequent sections of the protocol, the final decision was to define "Comparator" as "if included, placebo or guideline-based treatment", hence if the study reported a control group that received one of these, we gathered such data, reported and discussed it in the review (the above refers also to the entry 21.)

2) (v3) in entry 16. "Searches" the line "Search dates" the range "05.04.2023-26.04.2023" was initially intended to represent the period of article screening when the idea of the review was first conceived, but this turned out impossible to modify later; due to summer holiday absence of the review authors the ultimate date of data acquisition beginning was set to 15th October 2023 and the last screening for the new articles was done at 31th December 2024; hence the actual search dates should be "15.10.2023-31.12.2024" (ultimately, the following statement "No publication date restriction" imposes that all the articles published before 31.12.2024 would be considered).

SUPPORT

Sources
This review receives no financial support.

Sponsor
N/A

Role of sponsor or funder
N/A

Rationale
Atopic dermatitis (AD) is a chronic and recurrent inflammatory skin disease characterized by intense pruritus and eczematous lesions that appear in specific areas of the body (depending on the patient's age). Most patients report a personal or family history of atopy. Global prevalence of AD ranges from 10% to 20% in children and from 2% to 8% in adults. Traditionally, AD was classified into infantile (<2 years), childhood (2-12 years), and adolescent/adult (>12 years) types. Each type has a distinct morphology and anatomical distribution of skin lesions.

Recently, AD in subjects ≥60 years was acknowledged as separate variant of the disease. Comorbidities, polypharmacy, and increased susceptibility to infections in elderly patients require a reasonable balance between the effectiveness and safety of therapy.

Standard treatment of AD include patients education, avoidance of exacerbating factors, use of moisturizers and topical anti-inflammatory drugs. In more severe cases, additional treatment options such as phototherapy and biologics are available. The increased risk of complications restricts the use of cyclosporin A (CsA) or other immunosuppressive medications in elderly patients with AD.

Dupilumab is a fully human monoclonal antibody that inhibits α-subunit of the interleukin (IL) 4 receptor (a common target of IL-4 and IL-13) and the signaling of crucial cytokines responsible for the promotion of the TH2-dependent immune response. It was shown to be effective in treatment of moderate-to-severe AD in adults with rapid responses and scarcity of adverse effects.

However, no comprehensive synthesis of the available evidence exists for the group of AD patients ≥60 years of age. Therefore, the objective of this work was to systematically review data on dupilumab efficacy and safety in AD in elderly patients.

Objectives
The aim of this work was to systematically review data on dupilumab efficacy and safety in elderly AD.
PICO: Study participants are patients ≥60 years of age with AD (P) treated with dupilumab (I). The alternative intervention, if included, is placebo or guideline-based treatment (C). Treatment results are reported as improvement in quantitative measures of AD severity and the appearance of adverse reactions (O).

Eligibility criteria

Only articles written in English included.

Article inclusion criteria were determined according to PICO(S). Study participants are patients ≥60 years of age with AD (P) treated with dupilumab (I). The alternative intervention, if included, is placebo or guideline-based treatment (C). Treatment results are reported as improvement in quantitative measures of AD severity and the appearance of adverse reactions (O). The review of dupilumab effectiveness includes randomized controlled trials (RCTs), retrospective and prospective intervention studies (S). Case reports and case series would be included but discussed only regarding adverse reactions to dupilumab.

Information sources (v3)

Articles published in PubMed, Embase and Scopus databases before December 31, 2024.

(v4) An additional targeted gray literature search was carried out to minimize publication bias. The search included conference materials indexed in Embase as well as trials available at ClinicalTrials.gov and the World Health Organization International Clinical Trials Registry Platform. Google Scholar was searched to capture studies published in non-indexed journals.

Search strategy

Search query: "dupilumab AND (“atopic dermatitis” OR eczema) AND (elderly OR age OR old OR geriatric)". Additional manual review of the cited literature in the previously found articles.

Study records

Data management

All the records and data will be stored in an Excel file.

Selection process

Two independent reviewers would be responsible for the selection of studies for each phase of the review. In case of any conflicting assignments the third reviewer will make the final decision after a discussion.

Data collection process

Two independent reviewers would be responsible for the collection of the data from articles qualified for the review, i.e., data collection would be done in duplicate. In case there is any discrepancy between acquired information, the third reviewer would assess and extract the original data from the source article.

Data items

(1) First author, (2) study type, (3) year of publication, (4) country, (5) number of participants (male/female), (6) inclusion criteria, (7) patients age, (8) regimen and duration of dupilumab treatment, (9) initial severity of AD, (10) dupilumab’s efficiency, (11) adverse reactions, (12) onset of the disease, (13) clinical phenotype, (14) atopic comorbidities, (15) previous treatment.

If any data was available for few groups or was reported for few time points, all such data would be acquired and reported in the review.

Outcomes and prioritization

The outcomes are treatment results (reported as improvement in quantitative measures of AD severity) and the appearance of adverse reactions.

Because there is a number of measures available for the assessment of severity of AD, EASI score was selected as the main measure of improvement in this review. The rationale is that it is used in most of the studies and it covers both the area and the severity of skin lesions.

Notably, EASI scale omits subjective symptoms like itch or sleep disturbances – these are covered in SCORAD scale – however, SCORAD is rarely used in studies that investigate biologics.

The effect measures would be directly reported from the original source without any modifications.

In case there was any missing data or lacking information this would be clearly stated in the review and properly discussed.

The quality of the publications will be assessed with The National Institutes of Health (NIH) quality assessment tool.

Risk of bias in individual studies

The assessment of the risk of bias will be done with predefined tools – RoB2 (for randomized controlled trials) and ROBINS-I (for non-randomized intervention studies). The procedure will be done at the study level.

Data synthesis

The qualitative synthesis of the data is planned. This will include full presentation of the results of the review in the tables and thorough discussion of the outcomes with interpretation of: study types, quality, number of participants, subjects demographics (sex and age), measures of atopic dermatitis severity and improvement (EASI, SCORAD, BSA, NRS, DLQI), results of laboratory testing of atopic dermatitis severity biomarkers (if available), the adverse reactions to dupilumab (type and frequency). Information from case reports and series would be used for discussion over rare but hypothetically specific complications of dupilumab.

(v2) Furthermore, the quantitative synthesis (meta-analysis) of the suitable data is also planned. This includes evaluation of the absolute and relative improvement in EASI, P-NRS and DLQI (and other clinical scores, if suitable). A threshold for heterogeneity was set for I^2 >50% and Cochrane Q $P < 0.10$. In case of low heterogeneity among the studies, the fixed-effects model will be used for analysis. Otherwise, the random-effects model will be used. Subgroup analysis will be done with respect to different categorical factors such as study quality, country, study type and other relevant categories. Sensitivity analysis with consecutive exclusion of every single study from the analysis will be done. Meta-regression will be conducted to identify potential predictors of dupilumab effectiveness. P value < 0.05 will be

considered significant unless stated otherwise. All analyses will be done in R software (version 4.4.1; R Core Team, 2024).

Meta-bias(es)

(v2) Publication bias will determined with the visual assessment of the funnel plots and Egger's test.

Confidence in cumulative evidence

The confidence in cumulative evidence would be summarized in the discussion together with the interpretation of data synthesis.

(v2) The main quantitative results of the analysis will be presented as mean change with 95% confidence interval. The magnitude of standard error (SE) for each study and the overall estimate will be provided.
